# Identification of Cerebral Artery Stenosis Using Bilateral Photoplethysmography

**DOI:** 10.1155/2018/3253519

**Published:** 2018-03-19

**Authors:** Hyun Goo Kang, Seogki Lee, Han Uk Ryu, Youngsuk Shin

**Affiliations:** ^1^Department of Neurology, Chosun University School of Medicine and Hospital, No. 375 Seosuk-dong, Dong-gu, Gwangju 501-759, Republic of Korea; ^2^Department of Thoracic Surgery, Chosun University School of Medicine and Hospital, No. 375 Seosuk-dong, Dong-gu, Gwangju 501-759, Republic of Korea; ^3^Department of Neurology, Chunbuk National University School of Medicine and Hospital, San 2-20, Geumam-dong, Deokjin-gu, Jeonbuk 561-180, Republic of Korea; ^4^School of Information and Communication Engineering, Chosun University, No. 309 Pilmun-daero, Dong-gu, Gwangju 61452, Republic of Korea

## Abstract

Cerebral artery stenosis is currently diagnosed by transcranial Doppler (TCD), computed tomographic angiography (CTA), or magnetic resonance angiography (MRA). CTA exposes a patient to radiation, while CTA and MRA are invasive and side effects were related to contrast medium use. This study aims to provide a technique that can simply discriminate between people with normal blood vessels and those with cerebral artery stenosis using photoplethysmography (PPG), which is noninvasive and inexpensive. Moreover, the measurement takes only 120 seconds and is conducted on the fingers. The technique projects the light of a specific wavelength and analyzes the pulse waves which are generated when the blood passes through the blood vessels according to one's heartbeat using the transmitted light. Normalization was performed after dividing the extracted pulse waveform into windows, and maximum positive and negative amplitudes (MPA, MNA) were extracted from the detected pulse waves as features. The extracted features were used to identify normal subjects and those with cerebral artery stenosis using a linear discriminant analysis. The study results showed that the recognition rate using MPA was 92.2%, MNA was 90.6%, and combined MPA + MNA was 90.6%. The technique proposed is expected to detect early stage asymptomatic cerebral artery stenosis and help prevent ischemic stroke.

## 1. Introduction

Photoplethysmography (PPG) is a technology that presents the pulse wave generated by the blood passing through the blood vessels along with the heartbeat and is measured by extracting the transmitted light after projecting the light of a specific wavelength. The measurement of a biosignal using PPG is noninvasive, and it is possible to measure various signals including respiration, heart rate, vasomotor activity, and saturation by pulse oximetry (SPO_2_) using just one probe [1–4]. PPG can be used to evaluate atherosclerosis [5, 6], arterial stenosis [7–10], arterial properties [2, 11–13], hypertension [10, 12], diabetes mellitus [14], and cardiovascular risk factors [15]. PPG has been widely used to study cardiovascular function. PPG is a very effective method measuring the blood volume of each heartbeat in the body using the characteristics of the light. It enables the measurement of the subtle changes in blood volume of the arterioles and capillary vessels in the tissue along the systole-diastole cycle of the heart by using the changes in the transmittance of light even when the absorbance of a material is unknown.

Cardiovascular function is closely related to the brain, the top-level organ regulating all the body's functions. The brain requires high energy to maintain its vigorous metabolism, and energy is delivered to the brain by the blood. The blood is then delivered to every area of the body through the blood vessels. Ischemic stroke (cerebral infarction) indicates a cerebrovascular disease induced by energy depletion in the brain due to the insufficient blood supply to the brain caused by the abnormal blood vessel to the brain that damages the brain tissues and causes abnormal brain function. Cerebral artery stenosis may induce hemodynamic disturbance in the flow of the blood to the brain that can create blood clots in the narrowed blood vessel. Therefore, it is an important risk factor causing ischemic stroke.

The cerebral artery is divided into large arteries (e.g., cerebral artery) and small perforating arteries that diverge from the large artery. The carotid and vertebral arteries are important large arteries that supply blood to the brain from the aorta. Since stenosis slowly progresses in the large arteries, the cerebral artery does not show any symptoms until the blood vessel is occluded. When the blood vessel is completely occluded due to stenosis, blood is not supplied to brain parenchyma. Consequently, cell death occurs due to ischemia and symptoms associated with the necrosis of these cells and tissues occur. Ischemic stroke indicates these phenomena. Since necrotic brain cells do not regenerate, most people who experience ischemic stroke suffer permanent disability. Therefore, it is very important to prevent ischemic stroke and identify its curable risk factors such as cerebral artery stenosis.

Magnetic resonance angiography (MRA), computed tomographic angiography (CTA), carotid ultrasonography (CUS), and transcranial Doppler (TCD) are representative methods used to confirm cerebral artery stenosis. However, the majority of cases of cerebral artery stenosis are asymptomatic, as stated above, so it is recommended that preventative measures should be taken via periodic examinations. Most of the stated tests are expensive and have a risk of side effects due to the use of contrast medium. In addition, CUS can detect carotid artery stenosis at the neck and TCD has a poor temporal window because it cannot visualize the area when the temporal bone is too thick.

The objective of this cardiovascular function study was to evaluate the correlation between cerebral artery stenosis and the PPG signal reflecting the characteristics of microvessels in the tissues according to the cardiac contraction and relaxation cycle. If there is a correlation between the PPG signal and cerebral artery stenosis, it is expected that we can periodically screen for the presence of cerebral artery stenosis using a noninvasive, side-effect-free, and inexpensive technology instead of the known invasive and expensive methods. In addition, the advantage of PPG screening is that it can simultaneously check for stenosis of the intracranial artery and the carotid artery rather than that of limited blood vessels. People can easily notice that a more thorough blood vessel test is necessary when abnormalities are observed in the screening test. Moreover, if the location and degree of cerebral artery stenosis are found on a thorough medical examination, the stenosis can be treated by using drug therapy in the early stage and managed systematically by exercise. The results of this study will help clinicians control the occurrence of ischemic stroke, which burdens society.

## 2. Material and Methods

**Figure 1:**
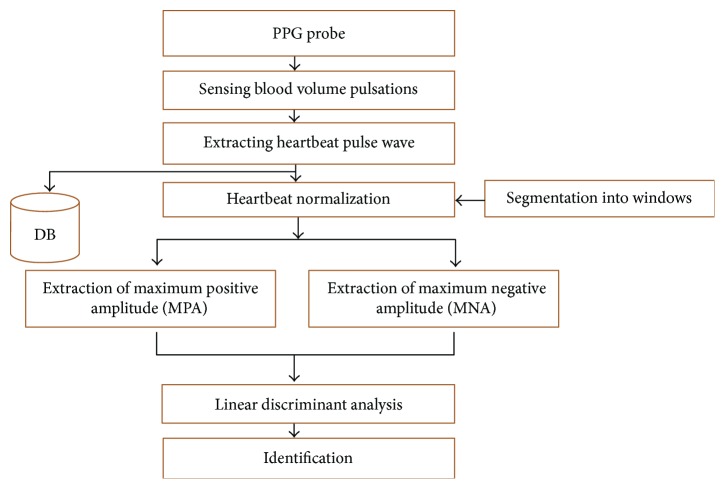
Flow diagram of the proposed screening method.

The PPG waveform amplitude was used to extract the characteristics of cerebral artery stenosis between the neck and the brain. Previous results showed that the PPG pulse wave amplitude was proportionate to the vessel distensibility under highly variable heartbeat conditions [16]. Data collection and preprocessing were conducted as a preparation step to extract the PPG waveform amplitude's characteristics. After preprocessing, the amplitude was transformed as a normalized pulse wave to compare the subjects' amplitudes. The characteristics of the amplitudes were extracted from the normalized pulse waves. The extracted features were analyzed using a linear discriminant analysis method and used to differentiate normal people from patients with cerebral artery stenosis. A schematic diagram of the proposed technique is shown in Figure 1.

### 2.1. Data Collection

In recent years, MRA has been increasingly used as a noninvasive imaging method for evaluating intracranial cerebral artery atherosclerosis [17], intracranial cerebral artery stenosis [18], internal carotid artery (ICA) stenosis, or middle cerebral artery (MCA) stenosis [19–21]. Stenosis of the cerebral artery on MRA is frequently observed in the ICA and MCA [18–21]. Therefore, the detection of cerebral artery stenosis within ICA or MCA appears greatly accomplished via a noninvasive method such as MRA.

Our study subjects were divided into a treatment group and a control group. The treatment group included outpatients with cerebral artery stenosis or ischemic stroke detected on brain MRA who consented to participate in the study. The control group included patients without cerebral artery stenosis who consented to participate in the study. The medical records and brain MRA scans were reviewed and determined by H.G.K (a neurologist). A total of 64 research subjects were included: 32 in the treatment group and 32 in the control group. The study subjects received an explanation of the PPG test method, provided written consent, and underwent PPG measuring. This study was conducted after receiving approval from the Chosun University Hospital Medical Ethics Review Committee (CHOSUN 2016-06-017).

**Figure 2:**
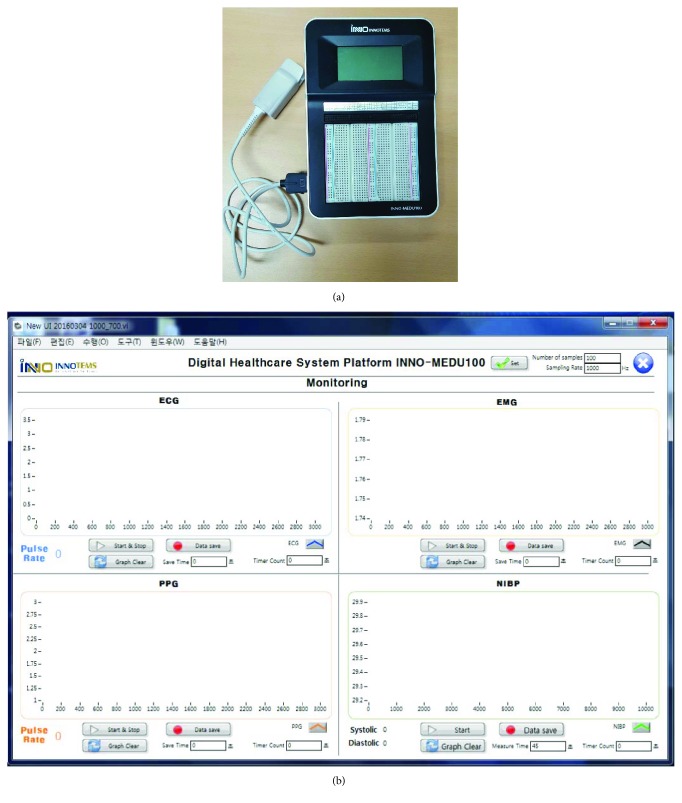
(a) Measurement device. (b) Screenshot of the photoplethysmography (PPG) measurement software.

Blood oxygen saturation measurement sensors were connected to an INNO-MEDU 100, a biological signal measurement system development kit based on medical grade sensors (INNOTEMS Co. Ltd., Korea). PPG probes were mounted on the index fingers of a study subject in a seated position. The pulse wave was measured for 2 minutes, and data were stored in a PC wirelessly. The PPG measures the pulse wave using the differences in light attenuation in the blood using the photodetector detecting the decay of transmitted light irradiated from a light-emitting diode light to the capillary vessel in an index finger. Figures 2(a) and 2(b) show screenshots of the measurement instrument and the PPG measurement software used in this study.

### 2.2. Preprocessing

**Figure 3:**
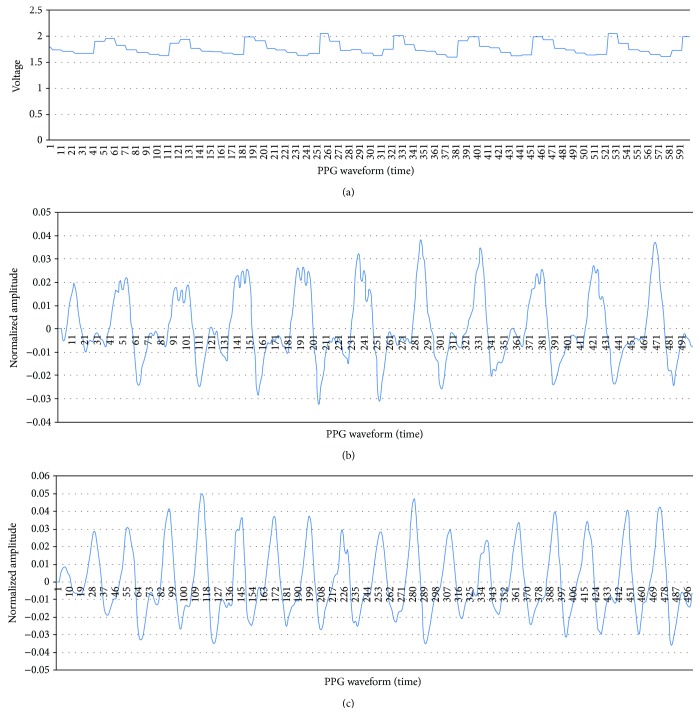
(a) Original PPG signal acquired from the measurement device. (b) Resampled PPG signal. (c) Optimized PPG signal.

It is necessary to resample a certain number of waveform data within a set timeframe to compare the magnitude of the subjects' waveform amplitudes measured by the PPG pulse wave. The resampling of this study was conducted by extracting 66,000 data points during 1 minute (60 seconds) from the initial waveform data points (1,200,000) extracted at a 1 KHz sampling rate during 2 minutes (Figure 3(a)). After resampling in 66,000 data points, the resampled data size reduced to 449 KB from the initial waveform data size 10.3 MB. The reduced data size provides great advantages for fast processing time and a small memory size. The resampling data were designed to facilitate the extraction of the heart rate within the waveform. The extracted resampling data have an overlapped wave pattern in a single heartbeat (Figure 3(b)).

**Figure 4:**
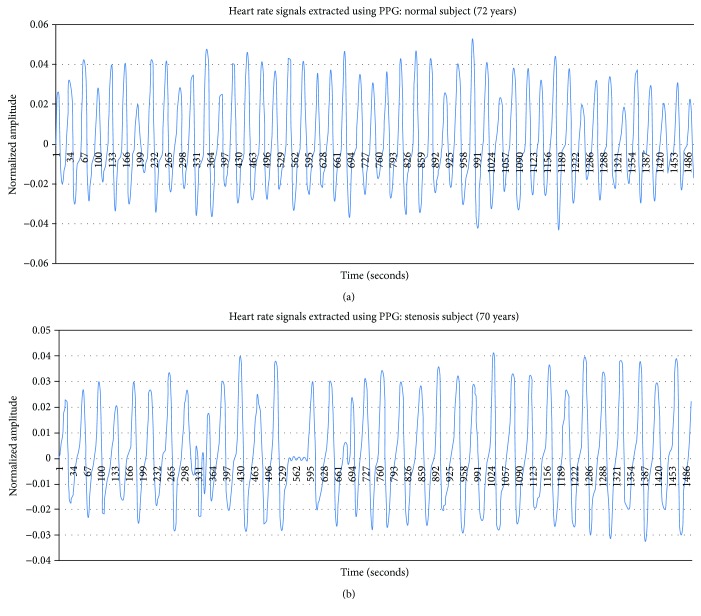
Pulse wave signals extracted from a normal subject and from a subject with cerebral artery stenosis after optimization sampling: (a) normal subject and (b) cerebral artery stenosis subject.

Therefore, the second step of the resampling procedure should be conducted to remove it and produce the optimized sample pulse wave (Figure 3(c)). The optimization sampling was used to design the sampling interval to make the pulse wave include mean heart rate per minute. In this study, sampling was conducted at 23 intervals.  Figure 4 shows the pulse waves extracted from the control and treatment groups after the optimization sampling. The results revealed that the amplitude of the normal subject (Figure 4(a)) was larger than that of the patient with cerebral artery stenosis (Figure 4(b)).

### 2.3. Feature Extraction

Multiple optimally sampled pulse waves were normalized to reduce the changes in the pulse wave signals that showed variations. Pulse wave normalization was conducted as follows:

(1) $$\begin{array}{c} w=sqrt\left(\overset{N}{\underset{j=1}{\sum }}{\left(Y_{ij}\right)}^{2}\right),\\ Y_{i}^{*}=\frac{Y_{i}}{w}. \end{array}$$

*Y* = {*Y*_*i*_}_*i*=1_^*N*^ can be calculated where *Y* is the total number of measurements obtained from the subject and *N* is the number of subjects. Moreover, the pulse waves obtained from the same subject can be expressed as *Y*_*i*_ = {*Y*_*ij*_}_*j*=1_^*N*_*i*_^. Pulse wave (*Y*_*ij*_) indicates the *j*th pulse wave of the *i*th subject.

The normalized pulse wave of each subject was divided into multiple windows, and a representative pulse wave amplitude was calculated for the pulse wave of each window. The advantage of dividing the whole section into windows is the possible extraction of representative feature values from each similar pulse wave section in a continuous time scale. Moreover, it makes it possible to easily compare the magnitudes of the amplitudes between subjects in the same window. Two feature values were extracted for each window (i.e., maximum positive amplitude [MPA] and maximum negative amplitude [MNA]). MPA indicated the largest positive amplitude, and MNA indicated the largest negative amplitude in each window. Consequently, 120 features were extracted from 60 windows for each subject.

### 2.4. Identification

Normal subjects and those with cerebral artery stenosis were classified using a linear discriminant analysis (LDA) algorithm [22] based on the extracted MPA and MNA feature values. LDA is a method that uses a linear classifier and dimensionality reduction by mapping data along the main axis to maximize the class separation in a specific space. It can be used as a classifier when it is applied to previously extracted features. The basic principle of the LDA algorithm is to reduce the dimension of a feature vector for data by maximizing the ratio of inter- and intraclass scatter. Electrocardiography and oscillometric arterial blood pressure measurements were used to identify individuals using features of the heartbeats [23, 24]. These studies reported that the LDA technique is a successful classifier for the amplitude features of the heart rate. It was believed that the amplitude features extracted from the capillary blood vessels from the proposed PPG would be similar to the amplitude features of previous studies. Consequently, this study used the LDA algorithm to optimally classify normal subjects and those with cerebral artery stenosis using the MPA and MNA features extracted from each window.

When *N* = ∑_*i*=1_^*c*^*C*_*i*_ of the study subjects exists, *C* represents the classes to be classified and *N* is the number of PPG samples extracted from all subjects. This study classified the PPG samples of the 64 subjects into two groups. When a learning group is considered *Y* = {*Y*_*i*_^∗^}_*i*=1_^*C*^, each class is composed of *Y*_*i*_^∗^ = {*Y*_*ij*_^∗^}_*j*=1_^*C*_*i*_^ in the C class, where *Y*_*ij*_^∗^ indicates the features extracted from the PPG. *S*_WT_ and *S*_BT_ represent intra- and interclass scatter, respectively, as follows:

(2) $$\begin{array}{c} S_{WT}=\frac{1}{N}\overset{c}{\underset{i=1}{\sum }}\overset{c_{i}}{\underset{j=1}{\sum }}\left(Y_{ij}^{*}-\mu_{i}\right){\left(Y_{ij}^{*}-\mu_{i}\right)}^{T},\\ S_{BT}=\frac{1}{N}\overset{c}{\underset{i=1}{\sum }}\left(\mu_{i}-\mu \right){\left(\mu_{i}-\mu \right)}^{T}. \end{array}$$

*C* represents the number of classes, and *c*_*i*_ indicates the feature values in class *i*. The symbols *μ*_*i*_, *μ*, and *Y*_*ij*_^∗^ represent the mean of class *i*, the mean of all classes, and the *j*th feature value of class *i*, respectively. LDA finds a set of feature basis vectors described by *ψ* that maximizes the ratio between *S*_WT_ and *S*_BT_ of the training sample set [22] as follows:

(3) $$\begin{array}{c} \psi =\mathrm{arg max}\frac{\left|\psi^{T}S_{BT}\psi \right|}{\left|\psi^{T}S_{WT}\psi \right|}. \end{array}$$

When *S*_WT_ is nonsingular, the basis vectors *ψ* in (3) correspond to the first *M*(≤*N*) eigenvectors with the largest eigenvalues of *S*_WT_^−1^*S*_BT_. The feature representation based on the LDA is produced by projecting input features *Y*^∗^ onto the subspace spanned by the *M* eigenvectors *X* = *ψ*^*T*^*Y*^∗^.

## 3. Results

**Figure 5:**
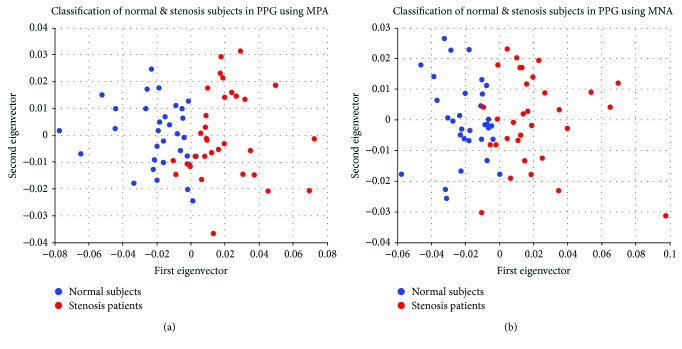
Classification results of normal subjects and those with cerebral artery stenosis using a linear discriminant analysis algorithm after applying the maximum positive amplitude (MPA) and maximum negative amplitude (MNA): (a) MPA features and (b) MNA features.

The classification results of the control group and treatment group were determined using the first eigenvector of the LDA algorithm and the MPA and MNA feature values. The MPA and MNA data extracted from both index fingers of the 64 study subjects were studied: 32 subjects in the control group (mean age, 59.8 ± 14.6 years) and 32 subjects in the treatment group (mean age, 62.7 ± 11.2 years). The feature values of MPA and MNA were composed of 60 data points each.  Figure 5 shows the classification results of the control and treatment groups using the first eigenvector and the second eigenvector in the LDA algorithm after applying each MPA and MNA feature.  Figure 5(a) reveals the results after application of the MPA features, while Figure 5(b) indicates the results after application of the MNA features. Regarding the decision boundary of the control and treatment groups, a patient was classified into the cerebral artery stenosis group when the first eigenvector from the LDA results was larger than 0 or into the normal group when it was smaller than 0.

The results showed that the recognition rate was higher when the features of both index fingers were used than when those of only one index finger were used. When the features of both index fingers were used, the recognition rate using MPA was 92.2%, while that using MNA was 90.6%.  Table 1 compares the recognition rate results when the features of the right index finger, left index finger, and both index fingers were used. When the combination of MPA and MNA was used for the classification, the recognition rate was 90.6%, which was not better than the results derived from the independent use of the MPA and MNA features.  Table 2 shows the sensitivity and specificity of the normal subjects and those with cerebral artery stenosis within the highest recognition rate. Sensitivity and specificity using the MPA features were 90.6% and 93.8%, respectively, while those using MNA features were 80% and 100%, respectively.

## 4. Discussion

Precision instruments such as MRA, CTA, CUS, and TCD are used to diagnose cerebral artery stenosis and have many advantages. However, diagnosing cerebral artery stenosis using PPG signals is advantageous because it is noninvasive, simple to use, and inexpensive. During the study period, 98 patients were asked to participate in this study. Of them, 31 patients refused to participate (control group: 19, stenosis group: 12) and 3 patients excluded due to poor image quality. Finally, 64 patients were enrolled and analyzed. The results of this study confirm the possibility of screening for cerebral artery stenosis using the PPG signal characteristics. The results of this study can be summarized as follows.

First, the results of the study indicated that the MPA and MNA features of the PPG signal may contribute to screening for early stage cerebral artery stenosis. In particular, MPA features are expected to show high classification accuracy, specifically 92.2%, for normal people and patients with cerebral artery stenosis. In contrast, MNA features revealed a 90.6% accuracy. Sensitivity and specificity analyses, a statistical method for evaluating performance, indicated that MPA features had a sensitivity of 90.6% and a specificity of 93.8%, while MNA features had a sensitivity of 80% and a specificity of 100%. A test's sensitivity represents its ability to correctly identify patients with a certain condition [25]. Regarding a medical diagnostic test's ability to diagnose a disease, sensitivity means the percentage of people with the disease. On the other hand, specificity refers to a test's ability to correctly identify patients without a specific condition. Regarding medical diagnostic tests, sensitivity means the ratio of correctly diagnosed healthy people without the disease. The results of this study showed that the classification using MPA features showed >90% accuracy sensitivity and specificity, suggesting that MPA features have high accuracy for differentiating subjects with cerebral artery stenosis from normal subjects. The MNA features were less effective at identifying patients with cerebral artery stenosis (80%) but very effective at detecting normal people (100%).

**Figure 6:**
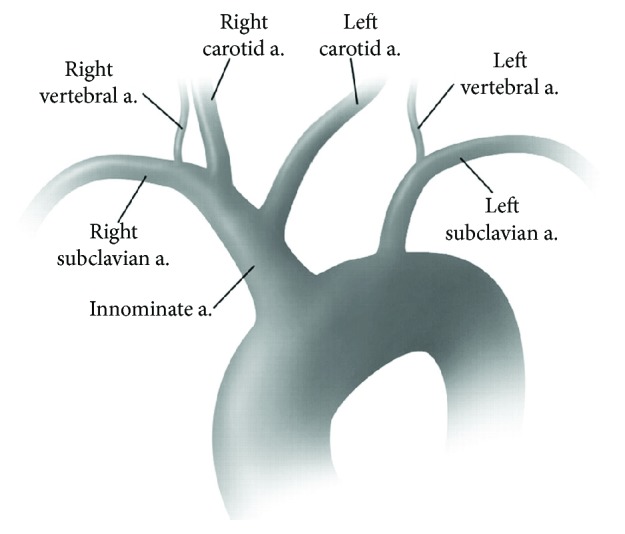
Anatomy of the common aortic arch branching patterns of the innominate, left carotid, and left subclavian arteries [26].

Second, using both index fingers showed the highest discrimination power for screening for early stage cerebral artery stenosis using the MPA and MNA features of PPG signals. In particular, the recognition rate of the right index finger was higher than that of the left index finger using both MPA and MNA features. This could be because of the anatomical characteristics that the left subclavian artery, delivering the blood from the heart to left arm, and common carotid artery, delivering the blood from the heart to the brain, emerge separately from the aortic arch on the left side of the body while the right subclavian artery, delivering the blood from the heart to the right arm, and right common carotid artery, delivering the blood from the heart to the brain, split from the innominate artery at the neck (Figure 6). In other words, we believe that the recognition rate was higher because the presence of cerebral artery stenosis could affect the ability of the right subclavian artery to move blood through the innominate artery to the right arm.

Third, discrimination between the normal group and the cerebral artery stenosis group could be successfully achieved using the first eigenvector of the LDA classification algorithm using the PPG signal. The decision boundary distinguishing normal people from people with cerebral artery stenosis was first eigenvector = 0; a positive first eigenvector indicated patients with cerebral artery stenosis, while a negative first eigenvector indicated normal people. The majority of errors generated in the recognition rate using the MPA and MNA features occurred in data near the decision boundary of a 0 value.

Fourth, the MPA and MNA features from PPG signals were determined as the important detector parameters for distinguishing normal people from those with cerebral artery stenosis. In particular, MPA features were considered the most important parameter with the best discriminatory power to detect the possibility of cerebral artery stenosis.

## 5. Conclusions

The objective of the study was to evaluate the correlation between cerebral artery stenosis and the PPG signal characteristics that could reflect the characteristics of the microvessels in the tissues according to the contraction and relaxation period of the heart. The study results showed that the MPA and MNA features of the PPG signals measured on the index fingers of both hands were important parameters for discriminating cerebral artery stenosis. MPA had particularly high discrimination power. Moreover, the right index finger better identified cerebral artery stenosis than the left index finger. One limitation of the study was its sample size. The study was conducted after patients provided informed consent, so it was challenging to secure a large number of study subjects. Therefore, the generalization of the study results should be made carefully. We plan to conduct a systematic study including more patients to identify a powerful and accurate marker for identifying patients with cerebral artery stenosis. To secure a large number of study subjects, we are designing multicenter linking neighboring hospitals and hospitals of overpopulation zone in the metropolitan area. The study results confirmed that PPG was a successful tool for screening for early stage cerebral artery stenosis. When a patient with cerebral artery stenosis is identified on the screening, the patient may receive clinical or drug treatment after a thorough examination. It is expected that the method presented here will play an important role in preventing cerebrovascular disease, which places a great burden on advanced society.

## Figures and Tables

**Table 1:** Experimental results based on the linear discriminant analysis (LDA).

Feature type	Experiment	Recognition rate (%)
Maximum positive amplitude	Left + right index finger	92.2
	Left index finger	78.1
	Right index finger	81.1

Maximum negative amplitude	Left + right index finger	90.6
	Left index finger	76.6
	Right index finger	79.7

**Table 2:** Sensitivity and specificity for the proposed technique in the best recognition rate.

	Feature type	Performance parameters	
		Sensitivity (true positive)	Specificity (true negative)
Proposed technique	MPA	90.6%	93.8%
	MNA	80%	100%

MPA: maximum positive amplitude; MNA: maximum negative amplitude.
